# Association of secondhand smoke exposure and health-related lifestyle behaviors among male university employees in Japan

**DOI:** 10.1038/s41598-023-40873-4

**Published:** 2023-09-11

**Authors:** Kaori Nakanishi, Chisaki Ishibashi, Seiko Ide, Ryohei Yamamoto, Makoto Nishida, Izumi Nagatomo, Toshiki Moriyama, Keiko Yamauchi-Takihara

**Affiliations:** Health Care Division, Health and Counseling Center, Osaka University, 1-17 Machikaneyama, Toyonaka, Osaka 560-0043 Japan

**Keywords:** Health care, Risk factors

## Abstract

Secondhand smoke (SHS) exposure causes various health problems associated with an unhealthy lifestyle. However, the lifestyles of individuals exposed to SHS have not been characterized extensively. Therefore, this cross-sectional study aimed to investigate the association between SHS exposure and lifestyle behaviors. The participants were 2379 healthy male employees at Osaka University who underwent health examinations. Physical and biochemical parameters and lifestyle behavior data were obtained from all the participants. Participants with SHS exposure had significantly higher body mass index, waist circumference, and serum levels of triglycerides and uric acid than that of those without SHS exposure. SHS exposure was significantly correlated with several lifestyle behaviors, including TV time, frequency of breakfast consumption and fried food consumption, vegetable and fruit intake, alcohol consumption frequency and daily alcohol intake, and smoking status. Thus, SHS exposure may be associated with an unhealthy lifestyle. The lifestyle behaviors of the smoke-excluded population were assessed further; however, SHS exposure was still associated with dietary and drinking habits. Since participants with SHS exposure are likely to have an unhealthy life and combined unhealthy lifestyle behaviors, the confounding effect of these factors should be considered when assessing the impact of SHS exposure on health.

## Introduction

The harmful effects of smoking on health are not limited to smokers and extends to those exposed to secondhand smoke (SHS). SHS contains hundreds of toxic chemicals and approximately 70 carcinogens. Thus, SHS exposure leads to approximately 880,000 deaths every year, globally^1–3^. SHS can cause various health problems including cardiovascular diseases, lung cancer, breast cancer, chronic obstructive pulmonary disease, sudden infant death syndrome, and depression^2,4–8^.

Like smoking, unhealthy lifestyles are associated with chronic diseases, known as non-communicable diseases (NCDs). More than 70% of all deaths globally are caused by NCDs including cancers, cardiovascular diseases, chronic respiratory diseases, and diabetes, accounting for 80% of NCD deaths^9^. Among the NCDs risk factors, lifestyle behaviors including smoking habits, physical inactivity, unhealthy diet, and excessive alcohol consumption, are considered extremely important based on their modifiability^10^. In addition to deaths caused by smoking, previous studies report that physical inactivity causes 3 million deaths, unhealthy diet causes 14 million deaths, and excessive alcohol consumption causes 2.3 million deaths each year^10^. Therefore, assessing and controlling lifestyle behaviors are necessary for NCD prevention and management.

Lifestyle behaviors of smoking and non-smoking populations have been reported to be different. Lifestyle in the smoking population was unhealthier than that in the non-smoking population^11^. As combined unhealthy lifestyle behaviors are more harmful to health than a single unhealthy lifestyle behavior^12^, detailed assessment of the lifestyle of individuals is necessary.

To the best of our knowledge, the lifestyles of individuals exposed to SHS have not yet been evaluated extensively. Thus, in the present study, we evaluated the association of SHS exposure and lifestyle behaviors using cross-sectional data from male university workers.

## Methods

### Study participants

This study included cross-sectional data collected from male Osaka University employees who underwent an annual health examination at the Osaka University Health and Counseling Center. The exclusion criteria for this study were as follows: (1) individuals with underlying health conditions, (2) individuals who were administered long term or frequent medication for at least 1 year before their health examination, (3) individuals with acute illness within the previous 2 weeks of the examination, and (4) individuals who did not answered the questionnaire on SHS exposure. In total, 2379 Japanese males were enrolled in this study. This study was conducted in accordance with the Declaration of Helsinki and the Ethics Guidelines for Clinical Research of the Ministry of Health, Labour and Welfare and the Ministry of Education, Culture, Sports, Science, and Technology. All experimental protocols in this study were approved by the Ethics Committee of the Health and Counseling Center, Osaka University, and written informed consent was obtained from all participants prior to participation in the study.

### Physical and biochemical parameters

Body mass index (BMI; body weight [kg] divided by height squared [m^2^]), waist circumference (WC) at the umbilical level, systolic blood pressure, and diastolic blood pressure were measured as physical parameters.

Serum was collected from participants after an overnight fast and stored at ≤ − 20 °C until it was assayed. The serum concentrations of aspartate aminotransferase, alanine aminotransferase, gamma-glutamyl transpeptidase (γ-GTP), creatinine (Cr), uric acid (UA), total cholesterol (TC), triglycerides (TG), high-density lipoprotein cholesterol (HDLC), fasting plasma glucose (FPG), and HbA1c were measured as biochemical parameters.

### Lifestyle behavior assessments

Information on SHS exposure and lifestyle behaviors of the study participants was obtained using questionnaires. All responses were reconfirmed through expert interviews with the trained nurses. SHS exposure was ascertained by a question “Are you regularly exposed to SHS indoors at home or the workplace?” and the answer was semi-quantified as 0 = no and 1 = yes. Lifestyle behavior questionnaires regarding physical and leisure activities, dietary habits, drinking habits, and smoking habits were asked as indicated below. Each answer was semi-quantified using the following scales, with larger number indicating an unhealthy lifestyle.

1. Exercise frequency: “How many days a week do you exercise?” on a five-point scale: 1 = 5–7 days a week, 2 = 3–4 days a week, 3 = 2 days a week, 4 = 1 day a week, 5 = 0 days a week.
2. Daily exercise duration: “How long do you exercise per day?” on a five-point scale: 1 =  > 120 min, 2 =  ≤ 120 min, 3 =  ≤ 60 min, 4 =  ≤ 30 min, 5 =  ≤ 10 min.
3. Time watching TV or videos: “How long do you watch TV or videos per day?” on a three-point scale: 1 =  < 30 min, 2 = 30–120 min, 3 =  > 120 min.
4. Breakfast consumption frequency: “How many days a week do you eat breakfast?” on a five-point scale: 1 = every day, 2 = 5–6 days a week, 3 = 3–4 days a week, 4 = 1–2 days a week, 5 = 0 days a week.
5. Lunch consumption frequency: “How many days a week do you eat lunch?” on a five-point scale: 1 = every day, 2 = 5–6 days a week, 3 = 3–4 days a week, 4 = 1–2 days a week, 5 = 0 days a week.
6. Dinner hour: “What time do you eat dinner on average?” on a four-point scale: 1 = by 7 PM, 2 = by 9 PM, 3 = by 11 PM, 4 = later than 11 PM.
7. Fried food consumption frequency: “How many days a week do you eat fried food?” on a three-point scale: 1 = 0–2 days a week, 2 = 3–4 days a week, 3 = 5–7 days a week.
8. Vegetable and fruit intake: “How many vegetables or fruits do you eat per day?” on a five-point scale: 1 = a large amount, 2 = above moderate, 3 = moderate amount, 4 = below moderate, 5 = a small amount.
9. Alcohol consumption frequency: “How many days a week do you drink alcohol?” on a five-point scale: 1 = 0 days a week, 2 = 1–2 days a week, 3 = 3–4 days a week, 4 = 5–6 days a week, 5 = every day.
10. Daily alcohol intake: “How much pure alcohol do you consume on a typical day when you are drinking?” on a three-point scale: 1 =  < 20 g, 2 = 20–40 g, 3 =  > 40 g.
11. Smoking status: “What is your smoking status?” on a three-point scale: 1 = never smoker, 2 = former smoker, 3 = current smoker.

### Statistical analyses

All statistical analyses were performed using STATA 14 (STATA Corp LLC, College Station, TX, USA). The distribution of continuous variables was assessed using the Shapiro–Wilk test. The Mann–Whitney U test was used to compare the two groups. Kendall’s rank correlation coefficient and multiple regression analysis using backward elimination method (p ≥ 0.2 removed) were used to analyze the variables. The chi-square test was used to compare the proportions of categorical values between the two groups. Statistical significance was set at *p* < 0.05.

## Results

### Characteristics of the study participants

The characteristics of the participants are presented in Table 1. Among the 2379 participants, 368 participants reported of being exposed to SHS: SHS exposure (+), and 2011 participants reported of not being exposed to SHS: SHS exposure (−). The median ages of the SHS exposure (+) and SHS exposure (−) participants were 37 (31–46) and 38 (32–45) years, respectively. In the SHS exposure (+) participants, BMI, WC levels (*p* = 0.013 and *p* = 0.017), and serum concentrations of UA and TG (*p* = 0.007 and *p* = 0.019) were significantly higher than that in the SHS exposure (−) participants. Serum Cr and HDL-C levels (*p* = 0.001 and *p* = 0.026) were significantly higher in the SHS exposure (−) participants than in the SHS exposure (+) participants.

**Table 1:** Characteristics of study participants.

	SHS exposure (+)	SHS exposure (–)	*p*
n	368	2011	
Age (years)	37 (31–46)	38 (32–45)	0.177
BMI (kg/m^2^)	23.4 (21.2–25.2)*	22.8 (21.0–24.9)	0.013
WC (cm)	82 (76–88)*	81 (75–87)	0.017
SBP (mmHg)	121 (112–130)	119 (111–129)	0.075
DBP (mmHg)	75 (68–82)	75 (68–82)	0.590
AST (IU/l)	21 (18–25)	21 (18–25)	0.769
ALT (IU/l)	21 (15–29)	21 (16–30)	0.683
γ-GTP (IU/l)	27 (19–40)	26 (19–40)	0.243
Cr (mg/dl)	0.83 (0.77–0.91)*	0.85 (0.78–0.92)	0.001
UA (mg/dl)	6.2 (5.5–7.1)*	6.1 (5.4–6.8)	0.007
TC (mg/dl)	196 (171–219)	196 (176–220)	0.250
TG (mg/dl)	89 (60–142)*	82 (58–120)	0.019
HDLC (mg/dl)	57 (48–68)*	59 (50–68)	0.026
FPG (mg/dl)	87 (83–92)	87 (83–92)	0.349
HbA1c (%)	5.2 (5.1–5.4)	5.3 (5.1–5.4)	0.960

Data are expressed as medians (interquartile range).

*BMI* body mass index, *WC* waist circumference, *SBP* systolic blood pressure, *DBP* diastolic blood pressure, *AST* aspartate aminotransferase, *ALT* alanine aminotransferase, *γ-GTP* gamma-glutamyl transpeptidase, *Cr* creatinine, *UA* uric acid, *TC* total cholesterol, *TG* triglycerides, *HDLC* high-density lipoprotein cholesterol, *FPG* fasting plasma glucose.

*p < 0.05 versus SHS exposure (–) participants.

### Association of SHS exposure with lifestyle behavior parameters

Table 2 presents the correlations between SHS exposure and lifestyle behaviors. SHS exposure had significant positive correlation with time spent watching TV or videos (τ = 0.043, *p* = 0.033), breakfast consumption frequency (τ = 0.098, *p* < 0.0001), fried food consumption frequency (τ = 0.051, *p* = 0.009), vegetable and fruit intake (τ = 0.057, *p* = 0.003), alcohol consumption frequency (τ = 0.058, *p* = 0.002), daily alcohol intake (τ = 0.106, *p* < 0.0001), and smoking status (τ = 0.237, *p* < 0.0001). These positive correlations suggest that SHS exposure is associated with an unhealthy lifestyle. For further analysis of the relation between SHS exposure and each parameter, multiple regression analysis was performed as presented in Table 3. Among the parameters, γ-GTP (β = − 0.0004, *p* = 0.024), Cr (β = − 0.159, *p* = 0.031), UA (β = 0.022, *p* = 0.003), TC (β = − 0.001, *p* = 0.039), TG (β = 0.0002, *p* = 0.039), FPG (β = 0.002, *p* = 0.038), breakfast consumption frequency (β = 0.014, *p* = 0.019), fried food consumption frequency (β = 0.026, *p* = 0.023), daily alcohol intake (β = 0.048, *p* = 0.003), and smoking status (β = 0.157, *p* < 0.0001) were observed as the influence factors of SHS exposure. There was no multicollinearity among these parameters.

**Table 2:** Correlations of secondhand smoke exposure with lifestyle parameters.

	τ	*p*
Exercise frequency	0.012	0.542
Daily exercise duration	0.003	0.897
Time watching TV or videos	0.043*	0.033
Breakfast consumption frequency	0.098*	< 0.0001
Lunch consumption frequency	0.036	0.073
Dinner hour	0.037	0.070
Fried food consumption frequency	0.051*	0.009
Vegetable and fruit intake	0.057*	0.003
Alcohol consumption frequency	0.058*	0.002
Daily alcohol intake	0.106*	< 0.0001
Smoking status	0.237*	< 0.0001

n = 1969, *p < 0.05.

**Table 3:** Parameters associated with secondhand smoke exposure.

	β	*p*
DBP	− 0.001	0.146
γ-GTP	− 0.0004*	0.024
Cr	− 0.159*	0.031
UA	0.022*	0.003
TC	− 0.001*	0.039
TG	0.0002*	0.039
FPG	0.002*	0.038
Breakfast consumption frequency	0.014*	0.019
Dinner hour	0.020	0.059
Fried food consumption frequency	0.026*	0.023
Alcohol consumption frequency	− 0.011	0.091
Daily alcohol intake	0.048*	0.003
Smoking status	0.157*	< 0.0001

Abbreviations are as in Table 1.

n = 1969, adjusted R^2^ = 0.117. *p < 0.05.

### Different lifestyle behaviors between participants with and without SHS exposure

**Figure 1:**
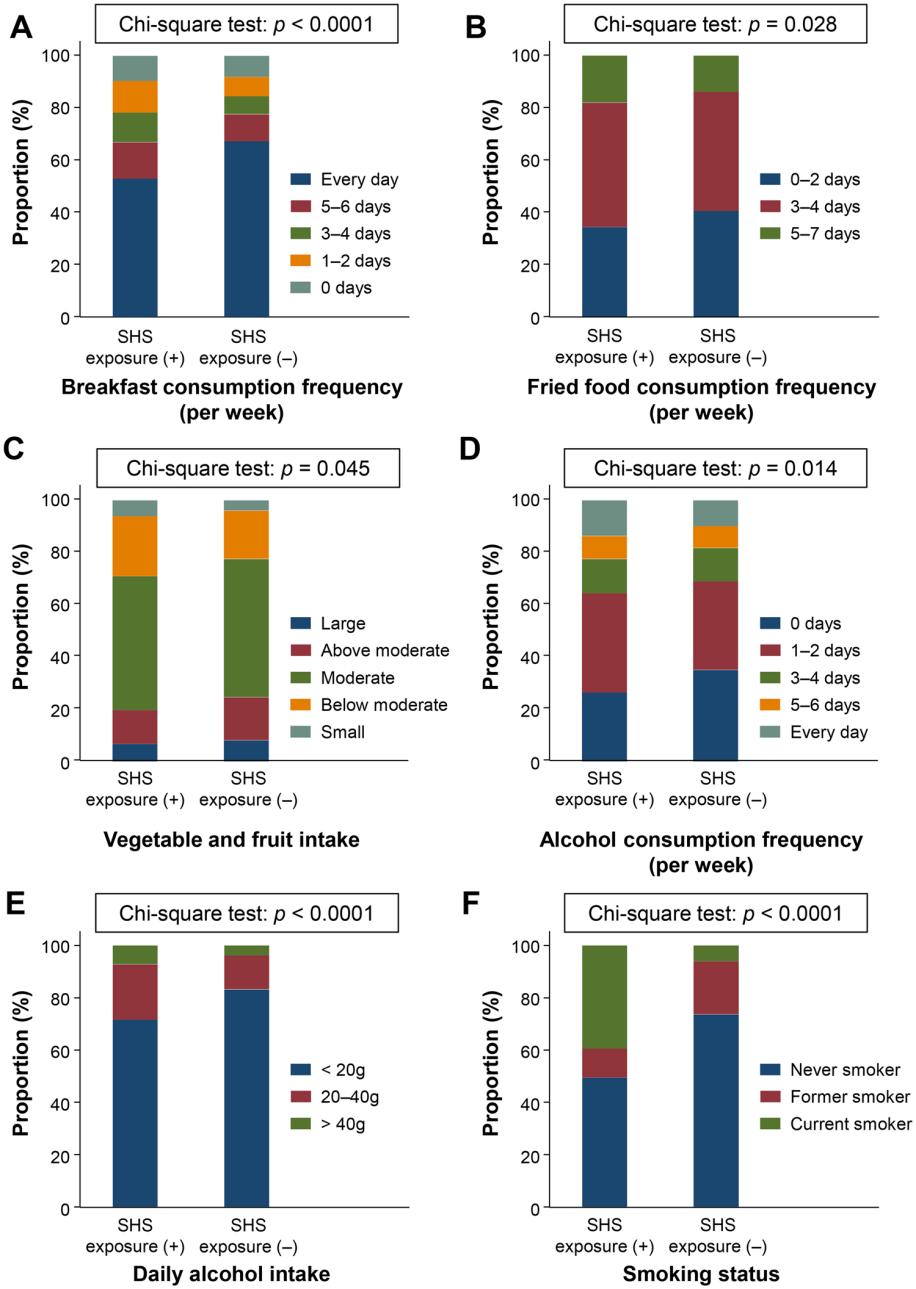
Differences of lifestyle behaviors among participants with and without secondhand smoke exposure. Comparison of lifestyle behaviors in SHS exposure (+) and SHS (–) participants. (**A**) Breakfast consumption frequency, (**B**) fried food consumption frequency, (**C**) vegetable and fruit intake, (**D**) alcohol consumption frequency, (**E**) daily alcohol intake, and (**F**) smoking status. The chi-square test was used to for between-group differences. Data are shown as the proportion of participants (%) who answered the lifestyle behavior questionnaire.

Since SHS exposure was related to several lifestyle behavior parameters, we assessed the differences in lifestyle between SHS exposure (+) and SHS exposure (−) participants. A comparison of the proportion of participants who answered the lifestyle behavior questionnaires among SHS exposure (+) and SHS exposure (−) participants is presented in Fig. 1. Significant differences were observed in the breakfast consumption frequency (*p* < 0.0001), fried food consumption frequency (*p* = 0.028), vegetable and fruit intake (*p* = 0.045), alcohol consumption frequency (*p* = 0.014), daily alcohol intake (*p* < 0.0001), and smoking status (*p* < 0.0001) between the two groups. Although long term TV viewing was correlated with SHS exposure, a significant difference was not observed between participants with and without SHS exposure (*p* = 0.094).

### SHS exposure associated with dietary and drinking habits in current smokers excluded population

Figure 1 shows that the prevalence of current smokers was high in the SHS exposure (+) participants. Since the smoking population is reported to have unhealthy lifestyles compared to the non-smoking population^11^, the increased prevalence of current smokers might influence the lifestyle of participants with SHS exposure. Therefore, we further assessed lifestyle behaviors among the study participants, excluding current smokers. The characteristics of smoke-excluded population are presented in Supplementary Table S1. As shown in Table 4, SHS exposure was significantly correlated with breakfast consumption frequency (τ = 0.055, *p* = 0.008), lunch consumption frequency (τ = 0.050, *p* = 0.019), fried food consumption frequency (τ = 0.060, *p* = 0.004), vegetable and fruit intake (τ = 0.041, *p* = 0.042), and daily alcohol intake (τ = 0.085, *p* = 0.0001). Significant differences were also observed in the proportion of breakfast consumption frequency (*p* = 0.018), lunch consumption frequency (*p* = 0.043), fried food consumption frequency (*p* = 0.014), vegetable and fruit intake (*p* = 0.028), and daily alcohol intake (*p* < 0.0001) between participants with and without SHS exposure. Even after excluding the smoking population, SHS exposure was associated with dietary and drinking habits.

**Table 4:** Correlations of secondhand smoke exposure with lifestyle parameters in current smokers excluded population.

	τ	*p*
Exercise frequency	− 0.015	0.445
Daily exercise duration	− 0.009	0.681
Time watching TV or videos	0.034	0.089
Breakfast consumption frequency	0.055*	0.008
Lunch consumption frequency	0.050*	0.019
Dinner hour	0.025	0.259
Fried food consumption frequency	0.060*	0.004
Vegetable and fruit intake	0.041*	0.042
Alcohol consumption frequency	0.021	0.289
Daily alcohol intake	0.085*	0.0001

n = 1730, *p < 0.05.

## Discussion

Health problems caused by SHS exposure have become a serious problem; therefore, many countries have implemented indoor smoking bans in public places, including restaurants and bars. However, more than 80% of people worldwide are still exposed to SHS^13^. In the present study, we examined physical, biochemical, and lifestyle behavioral parameters among participants with and without SHS exposure. SHS exposure (+) participants had significantly higher BMI and WC than SHS (−) participants. While exposure to SHS is reported to be associated with overweight/obesity in children, only a few studies have reported an association between SHS exposure and weight gain in adult^14–17^. Several mechanisms, including inflammation and oxidative stress, have been associated with SHS exposure and overweight/obesity^18,19^. In the SHS exposure (+) group, significantly increased TG and UA were also observed along with increased BMI. As TG and UA levels are associated with oxidative stress^20,21^, SHS exposure may have induced oxidative stress in the participants. However, these significant differences were not observed after excluding the smoking population (see Supplementary Table S1). It is suggested that active smoke affected inflammation and oxidative stress, and consequently enhanced these differences. Distinguishing the effect of passive smoke from active smoke is difficult in current smokers with SHS exposure. Thus, when assessing the pure effect of SHS exposure, current smokers should be excluded. In this study, information on SHS was limited to “exposed to SHS or not”, however, the duration of SHS exposure is also often used as the information on SHS. As duration of SHS exposure has been reported to be associated with severity of diseases and health problems^19,22^, these physical and biochemical differences might be clarified according to the duration of SHS exposure.

This study revealed that SHS exposure was associated with several lifestyle behaviors, including TV time, diet, alcohol consumption, and smoking habits. TV viewing, considered a sedentary behavior, is the most common leisure activity. As a sedentary lifestyle is associated with health problems, prolonged TV viewing has been reported to be associated with all-cause mortality, type 2 diabetes, and cardiovascular diseases^23–25^. Regarding dietary habits, breakfast consumption, fried food consumption, and vegetable and fruit intake were associated with SHS exposure. Breakfast is the most important meal of the day, which affects the physical and mental health^26–28^. Fried food consumption is also a risk factor for cardiovascular diseases, hypertension, and obesity^29^. Intake of vegetables and fruits is associated with a reduced risk of NCDs, including cancer, cardiovascular diseases, and chronic respiratory diseases^30^. Moreover, significant differences were observed in these dietary habits between participants with and without SHS exposure. Thus, the dietary habits of SHS exposure (+) participants are unhealthy compared to those of SHS exposure (−) participants. Excessive alcohol consumption is a well-known risk factor for various health conditions and chronic diseases^31^. The alcohol consumption frequency and daily alcohol intake was significantly different between participants with and without SHS exposure. Besides the influence of oxidative stress from SHS exposure, sedentary behaviors, unhealthy diet, and excessive alcohol consumption might also cause an increase in TG and UA levels in participants with SHS exposure^32–34^. A comparison of the smoking status revealed that the prevalence of current smokers was high among SHS exposure (+) participants. Thus, participants exposed to SHS are likely to inhale both active and passive smoke, which is even more unhealthy. Moreover, we assessed the association between SHS exposure and lifestyle behaviors in the smoke-excluded population. Even though the lifestyle behaviors of the smoking population were excluded, we found that participants with SHS exposure were likely to have unhealthy dietary and drinking habits. From these lifestyle behavior assessments, this study suggests that participants with SHS exposure live an unhealthy life and have combined unhealthy lifestyle behaviors.

Combined unhealthy lifestyle behaviors is a significant risk factor for all-cause mortality. A combination of unhealthy lifestyle behaviors shows a synergistic effect; therefore, an increased number of unhealthy lifestyle behaviors is associated with a higher risk of mortality^12,35,36^. Hence, these confounding factors should be assessed to evaluate the risk of SHS exposure. A previous study reported that adherence to body weight, diet, alcohol intake, and physical activity recommendations was associated with a lower risk of mortality in former smokers^37^. As participants with SHS exposure have combined unhealthy lifestyle behaviors, preventing the exposure to SHS and promoting a healthy lifestyle based on multiple aspects would be essential for maintaining individual health and reducing the risk of health problems.

This study had some limitations. SHS exposure was self-reported by the participants. The serum or urine cotinine is an established marker for SHS exposure^38,39^. Since self-reported SHS exposure might underestimate the intensity and frequency of SHS exposure, the association between SHS exposure based on cotinine levels and lifestyle behaviors should be assessed in future studies. Another limitation was that this was a single-center study with limited age range of subjects. At Osaka University, an indoor smoking ban on public places, except for several smoking rooms, has been implemented since 2004. Thus, the study participants might have been less exposed to SHS in the workplace compared to the participants in other institutions. Moreover, as the study participants were university employees, their background including age, educational level, and income might be different from other population. Since age and socioeconomic status have been reported to impact on lifestyle behaviors^40–42^, further study regarding different age range and socioeconomic status is needed to be performed.

In conclusion, we evaluated the association between SHS exposure and physical, biochemical, and lifestyle behavioral parameters in the present study. BMI, WC, serum TG, and UA levels were elevated in SHS exposure (+) participants. SHS exposure is associated with several lifestyle behaviors, including TV time, dietary habits, alcohol consumption, and smoking status. Moreover, participants with SHS exposure were more likely to have an unhealthy lifestyle than participants without SHS exposure. To the best of our knowledge, this is the first study to reveal lifestyle behaviors in participants exposed to SHS. While avoiding to expose SHS has been the main target for preventing the health problems caused by SHS exposure, the lifestyle behaviors among individuals exposed to SHS have not been discussed extensively. However, this study showed that participants with SHS exposure are likely to have an unhealthy life and combined unhealthy lifestyle behaviors. This combination of unhealthy lifestyle behaviors is expected to result in serious health problems. Therefore, comprehensive health promotion would be necessary to prevent NCDs and other health problems caused by SHS exposure.

### Supplementary Information

Supplementary Information.

## Data Availability

The authors confirm that the data supporting the findings of this study are available within the article.
