# Supplementary material for: Association of secondhand smoke exposure and health-related lifestyle behaviors among male university employees in Japan
Source: Sci Rep. 2023 Sep 11;13:13848. doi: 10.1038/s41598-023-40873-4 (PMC10495441; doi:10.1038/s41598-023-40873-4)
Supplement: Supplementary file 1 — Supplementary Information. [file 41598_2023_40873_MOESM1_ESM.docx]

**Supplementary Information**

**Association of secondhand smoke exposure and health-related lifestyle behaviors among male university employees in Japan**

Kaori Nakanishi, MD, PhD ^*^, Chisaki Ishibashi, MD, PhD, Seiko Ide, MD, PhD, Ryohei Yamamoto, MD, PhD, Makoto Nishida, MD, PhD, Izumi Nagatomo, MD, PhD, Toshiki Moriyama, MD, PhD, Keiko Yamauchi-Takihara, MD, PhD

**Supplementary Table S1. Characteristics of study participants excluding smoking population.**

|  | SHS exposure (+) |  | SHS exposure (–) |  | *p* |
| --- | --- | --- | --- | --- | --- |
| n | 223 |  | 1889 |  |  |
| Age (years) | 37 (30–47) |  | 38 (32–45) |  | 0.302 |
| BMI (kg/m^2^) | 23.2 (21.1–25.0) |  | 22.8 (21.0–24.9) |  | 0.212 |
| WC (cm) | 82 (75–87) |  | 81 (75–87) |  | 0.428 |
| SBP (mmHg) | 121 (112–130) |  | 119 (111–129) |  | 0.121 |
| DBP (mmHg) | 75 (68–81) |  | 75 (68–82) |  | 0.836 |
| AST (IU/l) | 21 (18–25) |  | 21 (18–25) |  | 0.921 |
| ALT (IU/l) | 21 (15–28) |  | 21 (16–30) |  | 0.196 |
| γGTP (IU/l) | 25 (19–38) |  | 25 (19–39) |  | 0.942 |
| Cr (mg/dl) | 0.83 (0.78–0.91) |  | 0.85 (0.78–0.92) |  | 0.067 |
| UA (mg/dl) | 6.2 (5.5–7.0) |  | 6.1 (5.4–6.8) |  | 0.125 |
| TC (mg/dl) | 198 (175–221) |  | 197 (176–220) |  | 0.616 |
| TG (mg/dl) | 81 (59–129) |  | 81 (58–119) |  | 0.629 |
| HDLC (mg/dl) | 58 (51–69) |  | 59 (50–69) |  | 0.849 |
| FPG (mg/dl) | 88 (83–92) |  | 87 (83–92) |  | 0.185 |
| HbA1c (%) | 5.2 (5.0–5.4) |  | 5.3 (5.1–5.4) |  | 0.187 |

Data are expressed as medians (interquartile range).

BMI, body mass index; WC, waist circumference; SBP, systolic blood pressure; DBP, diastolic blood pressure; AST, aspartate aminotransferase; ALT, alanine aminotransferase; γGTP, gamma-glutamyl transpeptidase; Cr, creatinine; UA, uric acid; TC, total cholesterol; TG, triglycerides; HDLC, high-density lipoprotein cholesterol; FPG, fasting plasma glucose.

We further examined the data among current smokers excluded population. The characteristics of smoke-excluded population are shown in Supplementary Table S1.
